# Stickler Syndrome Type 1 with Short Stature and Atypical Ocular Manifestations

**DOI:** 10.1155/2016/3198597

**Published:** 2016-11-28

**Authors:** Manisha Goyal, Seema Kapoor, Shiro Ikegawa, Gen Nishimura

**Affiliations:** ^1^Rare Disease Clinic, Department of Pediatrics, SMS Medical College, Jaipur, Rajasthan, India; ^2^Division of Genetics & Metabolism, Department of Pediatrics, Maulana Azad Medical College, New Delhi, India; ^3^Laboratory for Bone and Joint Diseases, RIKEN Center for Integrative Medical Sciences, Tokyo, Japan; ^4^Department of Paediatric Imaging, Tokyo Metropolitan Children's Medical Center, Fuchu, Tokyo, Japan

## Abstract

Stickler syndrome or hereditary progressive arthroophthalmopathy is a heterogeneous group of collagen tissue disorders, characterized by orofacial features, ophthalmological features (high myopia, vitreoretinal degeneration, retinal detachment, and presenile cataracts), hearing impairment, mild spondyloepiphyseal dysplasia, and/or early onset arthritis. Stickler syndrome type I (ocular form) is caused by mutation in the* COL2A1* gene. Ptosis and uveitis are relatively rare ophthalmological manifestations of this syndrome. We report an Indian boy having 2710C>T mutation in* COL2A1* gene demonstrating short stature, ptosis, and uveitis with Stickler syndrome.

## 1. Introduction

Stickler syndrome (STL) or hereditary progressive arthroophthalmopathy (OMIM #108300) is a heterogeneous group of genetic collagen tissue disorders with incidence of about 1/7,500 to 1/9,000 newborns [1]. Autosomal dominant forms of STL include type I (membranous vitreous) [STL1] caused by mutation in the* COL2A1* gene, type II (beaded vitreous form by* COL11A1* mutation), and type III (nonocular by* COL11A2* mutation). Autosomal recessive forms include type IV (*COL9A1* mutation) and type V (*COL9A2* mutation). Mutations in the* COL2A1* gene constitute about 80–90% cases of STL1. STL1 is characterized by a variety of ocular features including high myopia, vitreoretinal degeneration, retinal detachment, and presenile cataract. Extraocular manifestations comprise midface hypoplasia with cleft palate or Pierre Robin sequence, hearing loss (both conductive and sensorineural) and mild spondyloepiphyseal dysplasia and/or precocious osteoarthritis [2, 3]. Herein we report an Indian boy with STL1 demonstrating c.2710C>T mutation in* COL2A1* gene with short stature, ptosis, and uveitis, hitherto unreported in published literature.

## 2. Case History

**Figure 1:**
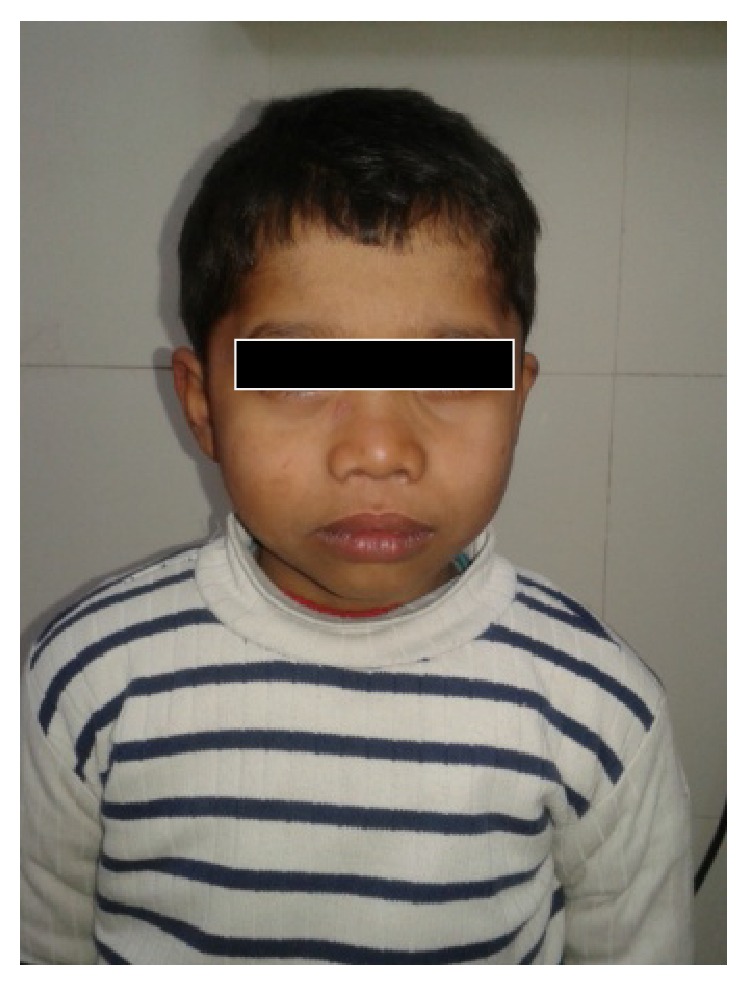
Facial features show midface hypoplasia, depressed nasal bridge, and thick lips.

**Figure 2:**
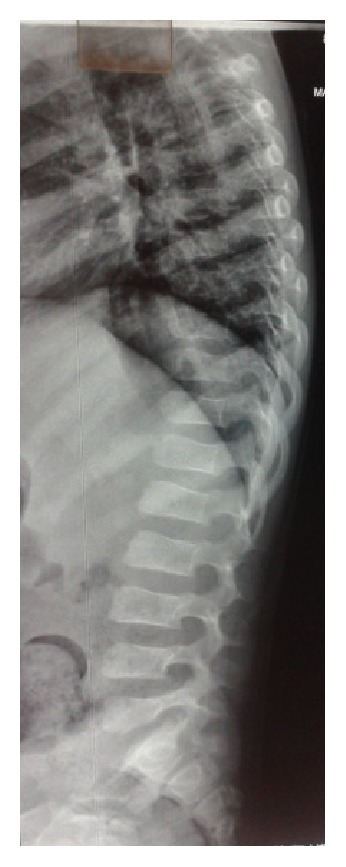
Lateral view of X-ray spine shows wedged shaped vertebral bodies with tongue-like anterior protrusion.

A seven-year-old boy was referred for evaluation of poor vision and hearing. He was sixth in birth order, born to consanguineous couple at term after normal vaginal delivery. He had bilateral congenital talipes equinovarus treated conservatively. He showed mild motor developmental delay with delayed walking. Poor vision and hearing were noted when he started attending school. Family history was unremarkable. On physical examination his weight, height, and head circumference were 22 kg (between 0 and −1 SD), 99 cm (<−3 SD proportionate short stature), and 47.8 cm (<3 percentiles), respectively. Upper segment (50 cm) to lower segment (49 cm) ratio was 1.02 and arm span was 95 cm. There was brachydactyly of fingers and toes. Facial features revealed midface hypoplasia, depressed nasal bridge, micrognathia, thick lips, and wide mouth. The left eye was smaller than the right along with eye ptosis and corneal opacity (Figure 1). Eye examination revealed myopia (right eye, −10.00 diopters; left eye, −12.00 D) and left chronic uveitis. Hearing evaluation suggested mild sensorineural deafness. Radiological examination revealed moderate platyspondyly. The vertebral bodies were dorsally wedged with tongue-like anterior protrusion evident at the thoracolumbar spine (Figure 2). The thorax was broad and short. The ilia appeared short and broad. Diffuse osteopenia was evident at the epiphysis of bilateral femora and tibiae which were normal in shape, apart from mild flattening of the distal femoral epiphyses. Metaphyseal broadening was found in the long bones, particularly of the proximal femora. The short tubular bones were mildly short and broad. Computerized tomography and ultrasonography eyes revealed hyperdensity in the vitreous cavity and multiple echoes in the vitreous one with echogenicity apparent from posterior aspect of lens till papilla in the center of vitreous one in left orbit. Ocular lens appeared mildly bulky and dense. Echocardiography and ultrasound of abdomen were unremarkable. In view of clinically suspected STL, genetic study was advised. After taking informed consent of the family, blood sample was sent for* COL2A1* gene study. DNA analysis revealed heterozygous mutation c.2710C>T (p.Arg904Cys) in exon 41 of* COL2A1* gene. It is a known pathogenic mutation that was already reported in STL patients [4].

## 3. Discussion

STL type 1 is a rare autosomal dominant condition with characteristic ophthalmological and orofacial features, hearing impairment, and mild spondyloepiphyseal dysplasia. The overall manifestations of the current case suggested the diagnosis of STL type 1. However, he showed a few unreported features. Significant short stature present in our patient has not been reported previously. In previous reports height was reported to be normal in most patients [5, 6]. Study by Beals reported slender extremities, long fingers, and normal height as body habitus of STL patients. In contrast, our patient showed a broad thorax and short fingers and toes [6].

Short stature despite involving both appendicular and axial skeleton maintained proportion in our case. The probable cause of short stature was major skeletal changes presented with spondylar dysplasia. The manifestation of the appendicular skeleton, including metaphyseal broadening and epiphyseal changes, was very mild. Features of midface hypoplasia, early onset high grade myopia, platyspondyly, and epiphyseal changes are compatible with differential diagnosis of acromesomelic dysplasia (AMD) (mesomelic shortening, platyspondyly, brachydactyly, prominent first toes, and midface hypoplasia) and spondyloperipheral dysplasia (SPD) (clubfeet, midface hypoplasia, myopia, platyspondyly, epiphyseal dysplasia, and brachydactyly). These conditions are excluded in our patients by absence of brachydactyly. Brachydactyly in AMD is usually more generalized and brachydactyly E-like changes developed in SPD in childhood [7]. Rose et al. evaluated the thoracolumbar spinal abnormalities in 53 patients from 24 families with Stickler syndrome. They found scoliosis in 34% cases, 74% endplate abnormalities, 64% Schmorl's nodes, 43% platyspondyly, and Scheuermann-like kyphosis in 43% [8]. Ballo et al. described a family with dominant-negative mutation in COL2A1 gene, with ocular problems and conductive deafness consistent with Stickler syndrome. In distinction to the classical form of Stickler syndrome, the affected persons had stubby digits and mild skeletal changes [9].

In our case, eye findings were high myopia, vitreous changes, corneal opacity, uveitis, and ptosis. In STL1 eye findings include high myopia (>−3 diopters) that is nonprogressive and detectable in the newborn period, congenital or early onset cataract, congenital vitreous anomaly, and rhegmatogenous retinal detachment [3, 10]. Vitreous changes in our case were likely to represent prior vitreous hemorrhage. Gerth et al. reported bilateral dense preretinal and vitreous hemorrhage in a newborn with novel mutation in* COL2A1* gene [11]. Bilateral corneal opacities in Stickler syndrome have been described [12]. Ptosis and uveitis present in the index case have not been described earlier to the best of our knowledge.

Once the diagnosis is established, a coordinated multidisciplinary team approach is required to be followed in STL1 patients and families. Early identification of ocular and auditory abnormalities allows early treatment and prevention of complications. Correct diagnosis allows prognosis of and surveillance for skeletal complications and genetic counseling for affected families.
